# Hydrogen-Rich Water and Lactulose Protect Against Growth Suppression and Oxidative Stress in Female Piglets Fed *Fusarium* Toxins Contaminated Diets

**DOI:** 10.3390/toxins10060228

**Published:** 2018-06-04

**Authors:** Weijiang Zheng, Xu Ji, Qing Zhang, Wenchao Du, Quanwei Wei, Wen Yao

**Affiliations:** 1 Laboratory of Gastrointestinal Microbiology, Jiangsu Key Laboratory of Gastrointestinal Nutrition and Animal Health, College of Animal Science and Technology, Nanjing Agricultural University, Nanjing 210095, Jiangsu, China; zhengweijiang@njau.edu.cn (W.Z.); jixuchance@gmail.com (X.J.); zhangqingzee@163.com (Q.Z.); 2 Laboratory of Animal Reproduction, College of Animal Science and Technology, Nanjing Agricultural University, Nanjing 210095, Jiangsu, China; wencdu@163.com (W.D.); weiquanwei@njau.edu.cn (Q.W.); 3 Key Lab of Animal Physiology and Biochemistry, Ministry of Agriculture, Nanjing 210095, Jiangsu, China

**Keywords:** lactulose, hydrogen-rich water, *Fusarium* mycotoxin, growth suppression, oxidative stress, piglet

## Abstract

The objective of the current experiment was to evaluate whether hydrogen-rich water (HRW) or lactulose (LAC) could protect against the adverse effects of *Fusarium* mycotoxins-contaminated diet on the growth performance and antioxidant status in weaning piglets. A total of 24 individually housed female piglets were randomly assigned to receive four treatments for 25 days (six pigs/treatment): uncontaminated basal diet (negative control), mycotoxin-contaminated (MC) diet, MC diet + HRW (MC + HRW) and MC diet + LAC (MC + LAC). The plasma hydrogen levels before and after 2 h hydrogen-free water/HRW administration were detected at day 21, and the liver hydrogen levels were detected at the end of the experiment. Serum hormones related to appetite regulation, and serum and liver oxidant and antioxidant status were also measured at the end of the experiment. Results showed that both HRW and LAC treatments significantly attenuated the reduction of average daily gain (ADG) and average daily feed intake (ADFI) caused by *Fusarium* mycotoxins. LAC administration increased the hydrogen concentrations in plasma and liver. HRW treated group had higher plasma hydrogen levels than the MC group. Compared with the NC group, the MC group had significantly increased serum peptide YY (PYY) and cholecystokinin (CCK) levels. Interestingly, both HRW and LAC administrations had a lower reduced serum PYY and CKK levels. Most importantly, oral administration of HRW and LAC attenuated the *Fusarium* mycotoxins-induced oxidative stress. In conclusion, oral administration of hydrogen-rich water or lactulose could both protect against the growth reduction and oxidative damage caused by *Fusarium* mycotoxins.

## 1. Introduction

*Fusarium* mycotoxins are secondary metabolites produced by *Fusarium* species of fungi, which can contaminate animal feed ingredients. Deoxynivalenol (DON) and zearalenone (ZEN) are considered to be the most important *Fusarium* mycotoxins due to their high toxicity [1]. A global survey indicated that *Fusarium* mycotoxins DON and ZEN, respectively, contaminated 55% and 36% of feed and feed ingredients in the period 2004–2011 [2], which results in a large economic loss in the livestock industry. Among farm animals, pigs are particularly sensitive to *Fusarium* mycotoxins [3]. Ingestion of DON is usually associated with feed refusal, low weight gains, low feed efficiency, diarrhea, and organ damage in pigs [3,4]. DON also significantly alters functions of the intestinal tract, including decreasing villus surface area and altering the permeability of the intestinal tract in weaning piglets [5]. Treatment with ZEN leads to hyperestrogenism, precocious puberty, and reproductive disorders [1,3]. In many cases, DON and ZEN often simultaneously found in naturally *Fusarium* fungi contaminated feed. Therefore, the co-occurrence of mycotoxins may make their toxicity effects on pigs more complicated. The liver is one of the main organs for metabolism of toxic substances, and liver damage might occur when pigs consume overdoses of mycotoxins [6]. An increasing number of studies have demonstrated that oxidative stress plays a major role in the toxicity of *Fusarium* mycotoxins [7,8].

Hydrogen gas (H_2_), historically thought an inert and nonfunctional gas, is defined as a selective antioxidant in recent years. Molecular hydrogen can selectively neutralize toxic free radicals such as hydroxyl radicals and nitrite anion without disturbing physiological reactive oxygen species (ROS) [9]. Unlike common antioxidant reagents, hydrogen has a unique advantage that it can penetrate biomembranes and diffuse into cellular components [10]. As a result, hydrogen has been applied in many animal liver damage models through its anti-inflammatory, anti-apoptotic, and anti-oxidant properties [11]. Inhalation of H_2_ gas suppresses hepatic ischemia/reperfusion injury by reducing oxidative stress in mice [12]. Hydrogen-rich water (HRW) has also shown remarkable protective effects against obstructive jaundice [13] and chronic EtOH-induced [14] liver damage, possibly by activating antioxidant enzymes.

Supplementation of hydrogen-producing prebiotics is also a feasible way of providing functional hydrogen to animals and humans [11]. Lactulose, a nonabsorbable synthetic disaccharide consisting of fructose and galactose, has been proposed as an indirect antioxidant by mobilizing intestinal hydrogen production [15]. Lactulose possesses neuroprotective [16] effects against cerebral ischemia/reperfusion injury and anti-inflammatory [17,18] effects on dextran sodium sulfate-induced colon inflammatory, which could be ascribed to the hydrogen production produced during microbial fermentation of lactulose. Lactulose administration accelerates liver regeneration in a rat hepatectomy model by inducing hydrogen [19]. Previous strategies to mitigate the toxic effects of *Fusarium* mycotoxins in animal production involved mycotoxins adsorbent and mycotoxins-degrading enzymes and micro-organisms.

We hypothesize that both HRW and lactulose may protect weaning piglets against growth depression and liver damage caused by *Fusarium* mycotoxins contaminated diet by increasing hydrogen levels and activating the antioxidant capacity in the body.

## 2. Results

### 2.1. Hydrogen Concentrations in Plasma and Liver

**Figure 1:**
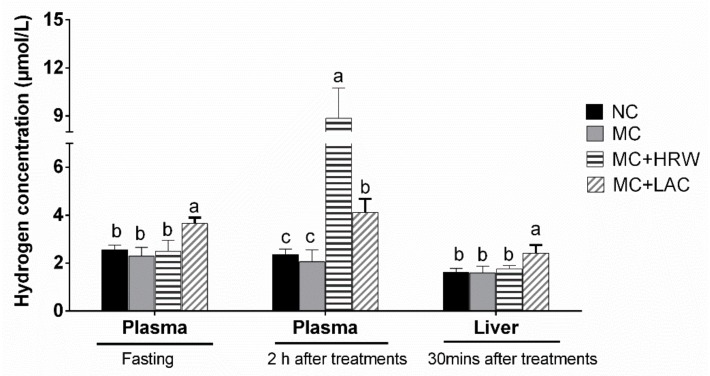
Effects of lactulose and hydrogen-rich water on plasma and liver hydrogen concentrations in female piglets fed *Fusarium* mycotoxins contaminated diets. Each column represents the mean hydrogen levels with five independent replicates, mean ± SD. a–c Letters above the bars indicate statistical significance (*p* < 0.05) among the four treatments. NC (negative control), basal diet; MC, *Fusarium* mycotoxins contaminated diet; MC + LAC, MC diet + lactulose treatment; and MC + HRW, MC diet + hydrogen-rich water treatment.

On day 21 of the experiment, plasma samples were collected for measuring hydrogen levels before and two hours after different administrations in four groups (Figure 1). Before administration, MC + LAC group had higher plasma hydrogen concentrations than the other three groups (*p* < 0.05); and no difference was found among the NC, MC and MC + HRW groups (*p* > 0.05). After two hours of oral administration, the levels of hydrogen in the MC + HRW group were greater than in the NC, MC and MC + LAC groups (*p* < 0.05). In addition, the MC + LAC group also had higher plasma hydrogen levels than the MC and NC groups (*p* < 0.05). No difference was found in the plasma hydrogen levels between the NC and MC groups (*p* > 0.05). At the end of the experiment (30 min after administration), hydrogen levels in the liver samples were further assayed (Figure 1). Results showed that liver hydrogen levels were not different among the NC, MC and MC + HRW groups (*p* > 0.05). However, the MC + LAC group had a higher hydrogen concentration in the liver than the other three groups (*p* < 0.05).

### 2.2. Growth Performance

Body weight (BW), average daily weight gain (ADG), average daily feed intake (ADFI), and feed to gain ratio (G:F) from three different time periods are summarized in Table 1. The BW on day 0, day 14 and day 25 were not different among the four treatments (*p* > 0.05). However, the ADG from days 0 to 14 and days 0 to 25 periods were considerably decreased by mycotoxins contaminated diet (*p* < 0.05). Compared with the MC group, both HRW and LAC groups had increased the average daily gain (ADG) (*p* < 0.05). *Fusarium* mycotoxins drastically reduced the ADFI in three different time periods from days 0 to 14, days 14 to 21 and days 0 to 25 (*p* < 0.05). However, HRW and LAC treatments increased the ADFI during the periods of days 0 to 14 and days 0 to 25 (*p* < 0.05). The ratio of G:F showed no difference among the treatments (*p* > 0.05).

### 2.3. Serum Levels of Appetite-Regulating Hormones

Table 2 shows that no difference was found in the serum ghrelin concentration among the four groups (*p* > 0.05). However, *Fusarium* mycotoxins contaminated maize resulted in higher serum peptide YY (PYY) and cholecystokinin (CCK) concentrations compared with the negative control diet (Table 2). Both MC + LAC and MC + HRW groups had lower serum PYY and CKK levels than the MC group (*p* < 0.05), and no difference was found when they were compared with the NC group (*p* > 0.05).

### 2.4. Oxidative and Antioxidative Status in the Serum and Liver

#### 2.4.1. Serum Oxidant Markers and Antioxidant Capacity

Indicators of serum oxidative and antioxidant status are presented in Table 3. The activities of serum catalase (CAT), total superoxide dismutase (SOD), CuZn-SOD, and Mn-SOD were not different among the NC, MC, MC + LAC and MC + HRW groups (*p* > 0.05). The serum total carbonyl and 8-hydroxydeoxyguanosine (8-OH-dG) levels in the MC group were significantly higher than in the other three groups (*p* < 0.05), whereas no difference was found among the NC, MC + LAC and MC + HRW groups (*p* > 0.05). Serum malondialdehyde (MDA) levels in the MC + LAC group were lower than the MC group (*p* < 0.05), while no difference was found among the NC, MC and MC + HRW groups (*p* > 0.05).

#### 2.4.2. Liver Oxidant and Antioxidant Capacity

Table 4 shows the liver oxidative and antioxidant status. Protein carbonyl, MDA, glutathione peroxidase (GSH-px), glutathione (GSH), GSSG, and reduced GSH levels were not different among the NC, MC, MC + LAC and MC + HRW groups (*p* > 0.05). The total carbonyl levels in the MC group were higher (*p* < 0.05) than in the MC + LAC and MC + HRW groups, whereas no difference was found between the NC and MC groups (*p* > 0.05). The inhibiting hydroxyl radical levels were not affected by *Fusarium* mycotoxins (*p* > 0.05), while the MC + HRW group had a higher inhibiting hydroxyl radical levels than the MC + LAC and NC groups (*p* < 0.05). The CAT levels in the MC + LAC and MC + HRW groups were lower than the MC group (*p* < 0.05), but no difference was found between the NC and MC groups (*p* > 0.05).

Total SOD, CuZn-SOD, and Mn-SOD were significantly impacted by the treatments. The total SOD activity in the MC group was significantly lower than in the NC, MC + LAC and MC + HRW groups (*p* < 0.05), and no difference was found among the NC, MC + LAC and MC + HRW groups (*p* > 0.05). The CuZn-SOD activity in the MC + LAC and MC + HRW was significantly higher than in the MC group (*p* < 0.05), and no difference was found between the NC and MC groups (*p* > 0.05). The Mn-SOD enzyme activity in the MC group was also significantly lower (*p* < 0.05) than in the NC group, and no difference was found between the MC + LAC and MC + HRW groups (*p* > 0.05).

## 3. Discussion

In the present study, we tested the hypothesis that administration of hydrogen-rich water or lactulose might protect against the adverse effects of *Fusarium* mycotoxins contaminated diet on growth performance and antioxidant status in female weaning piglets.

### 3.1. Hydrogen Concentration in Serum and Liver

Based on previous studies [20,21], we orally administrated hydrogen-rich water (10 mL/kg BW, 0.6 mM) and 500 mg/kg BW lactulose (two times/day) to piglets fed mycotoxins contaminated diet, respectively. To date, many researchers have reported the hydrogen concentrations in blood and tissues in vivo following administration of exogenous hydrogen treatment [22,23,24] or lactulose [16,19] using rodent models. However, the effects of hydrogen-rich water and lactulose on blood and liver hydrogen status in female piglets have never been addressed. A previous study showed that hydrogen concentrations reached the peak levels at 5 min after oral and intraperitoneal (IP) administration in rats [22], and highest hydrogen concentrations in the blood and tissues were observed at 30 min after the inhalation of hydrogen gas. Furthermore, breath H_2_ content rapidly reached to its maximal level 10 min after ingestion, and decreased to the baseline level within 60 min in adult volunteers [25]. Molecular hydrogen is produced continuously under normal physiological condition, primarily during the fermentation of nondigestible carbohydrates by bacteria in the large intestine. Recent evidence indicated that inhalation of hydrogen gas or HRW/hydrogen-rich saline or lactulose treatment could significantly increase the hydrogen concentrations in blood and tissues [22]. Here, plasma samples before and two hours after different oral treatments in piglets were collected. In addition, liver tissue was sampled after 30 min of administrations. Our data showed that *Fusarium* mycotoxins did not affect the hydrogen status in the plasma and liver (Figure 1). Considering the features of LAC and HRW, it is expected that lactulose administration caused a constant increase in plasma and liver concentrations of hydrogen (Figure 1), while HRW treatment only caused an increment in plasma samples after 2 h of administration.

Due to the high diffusion capability of H_2_, drinking hydrogen-rich water rapidly increased breath and tissues hydrogen content [22,23,24,25]. On the other hand, bacteria fermentation of lactulose will lead to a continual gas production, including hydrogen gas. Therefore, the difference of the hydrogen lease rate between the hydrogen-rich water and lactulose might contribute the discordance hydrogen concentrations between plasma and liver in the current study. Although higher hydrogen levels were observed in the LAC (plasma and liver) and HRW (plasma) treatments compared with the MC group, they do not represent the whole H_2_ production of HRW or LAC, and the dynamics of hydrogen gas after administration of lactulose or hydrogen-rich water in the blood and tissues in piglets is still unknown. Further studies are required to accurately determine the hydrogen concentrations in various tissues of piglets, which are very important for the application of hydrogen gas in animal production.

### 3.2. Growth Performance

Due to the dysfunctions of immune and digestive systems, weaning piglets is especially vulnerable to toxic substances, which may reduce growth performance and impair health condition. In a previous study, *Fusarium* mycotoxins contaminate maize (8.6 mg/kg DON and 1.2 mg/kg ZEN) was incorporated into a maize-based diet for piglets at 50% at the expense of control maize (equal to 4.3 mg/kg DON and 600 μg/kg ZEN). After five weeks of feeding, voluntary feed intake, and body weight gain were significantly decreased while the feed conversion ratio was not affected [26]. Cheng et al. [27] reported that feeding DON and ZEN contaminated diet (1.0 mg/kg DON and 250 μg/kg ZEN) significantly reduced the body weight, daily feed intake and ADG in weaning piglets. In our experiment, a similar result has been observed. In addition, symptoms include diarrhea, fever and refusal to feed were observed in piglets fed *Fusarium* mycotoxins contaminated maize. In this study, piglets were fed with relatively low *Fusarium* mycotoxins levels (501.56 μg/kg ZEN, 825.46 μg/kg DON and 272.54 μg/kg DON acetylated derivatives). It should be pointed out that the co-occurrence of mycotoxins in naturally *Fusarium* fungi contaminated feed may cause more intense toxicity [8]. It needs to be mentioned that the interactions between DON and ZEN may not be precisely predicted, since the possible presence of unknown *Fusarium* toxins may possess similar or even different modes of action, even if similar concentrations are applied [26].

Prebiotics are defined as “selectively fermented ingredients that result in specific changes in the composition and/or activity of the gastrointestinal microbiota, thus conferring benefits upon host health” [28]. In pigs, lactulose has been used as a prebiotic with beneficial effects cited as stimulating the growth of health-promoting bacteria in the gut and promoting growth performance [29,30]. Krueger et al. [31] reported that lactulose supplementation in periparturient sows reduced the length of pregnancy, and losses of their piglets and weaners, and increased daily weight gains and weight of weaners. A previous study also reported that 1% lactulose dietary supplementation in weaning piglets for two weeks enhanced feed intake, average daily gain and feed: gain ratio, which might be related to increased feed intake and improved gut integrity [29]. Furthermore, dietary lactulose addition (10 g/kg) was reported to increase the average daily weight gain after an oral enterotoxigenic *E. coli* K88 challenge in weaning piglets [21]. Here, our data showed that 500 mg/kg BW lactulose administration (two times/day) significantly attenuated the reduction of ADG and ADFI caused by *Fusarium* mycotoxins in weaning piglets. The ability to selectively change the composition of intestinal microbiota and/or intestinal integrity might be the underlying mechanisms of lactulose’s growth-promoting effects against *Fusarium* mycotoxins. However, recent evidence indicated that hydrogen gas may play a non-negligible role in lactulose’s beneficial effects [16,17,18,19]. In the present study, 10 mL/kg BW of HRW (two times/day) to weaning piglets was found to significantly enhance the ADG and ADGI in piglets fed *Fusarium* mycotoxins contaminated diet. To our knowledge, this is the first evidence that hydrogen-rich water could spare the harmful effect of *Fusarium* mycotoxins on growth performance of weaning piglets.

### 3.3. Serum Gut Appetite-Regulating Hormones Levels

Satiety hormones play a role in mediating *Fusarium* toxins-induced anorexia [4,32]. Gut satiety hormones peptide YY (PYY) and cholecystokinin (CCK) contributed to the feed refusal [33,34]. Feed refusal and higher plasma PYY and CCK levels were found in mice treated with DON, and direct administration of exogenous PYY or CCK also caused reduced food intake in mice [35]. In the current experiment, *Fusarium* mycotoxins increased the serum PYY and CCK levels compared with the NC group in weaning piglets. These findings are consistent with those studies which reported that both oral and IP administrations of DON in mice increased plasma CCK and PYY levels with concurrent food refusal [35,36]. Here, serum PYY and CKK levels were attenuated by both HRW and LAC administrations (Table 3). Ghrelin has been demonstrated to increase food intake, and promote weight gain and adiposity in rodents [37]. Although no difference was found in serum ghrelin levels in this study, a recent study demonstrated that orally administered hydrogen-rich water significantly increased gastric production of the ghrelin in mice [38]. This might suggest that satiety hormones are important for mediating the effects of lactulose and HRW on food intake and growth performance. Further studies are required to determine the possible underlying mechanisms.

### 3.4. Oxidative Stress

Studies have shown that oxidative stress plays an important role in the cytotoxic mechanism of mycotoxins [7,8]. The adverse effects of *Fusarium* mycotoxins on serum and hepatic oxidative stress vary with among studies, animal species, mycotoxins composition, and exposure time. For example, male broiler chickens exposed to 10 mg/kg DON for 17 days did not alter the total antioxidant status in plasma and glutathione peroxidase (GPx) levels in the liver [39]. No significant difference was found in serum total antioxidant status and GPx activity in male pigs after 14 days DON (4 mg/kg feeds) treatment [40]. However, higher plasma H_2_O_2_ and MDA levels were found in piglets fed the DON-contamination diet at 4 mg/kg for 30 days [41]. The high amount of ZEN in the contaminated diet may also contribute to oxidative stress in the liver or serum because purified ZEN can increase the MDA level in the serum and liver in piglets [42]. In addition, prepubertal gilts fed with diets contaminated with ZEN (0.5 and 2 mg/kg) had lower MDA, GPx, and SOD in the serum [43]. Combined with the serum biochemical variables (data not published), it is reasonable that piglets fed the MC diet had higher oxidative stress biomarkers (total carbonyl, lipid peroxidation, CAT, and 8-OH-dG) and lower antioxidant enzymes (total SOD, CuZn-SOD, and Mn-SOD) in the serum and hepatic samples.

Many studies have reported that the hydrogen gas exerts organ-protective effects through regulating oxidative stress, inflammation, and apoptosis [9]. Li et al. [44] observed that hydrogen saline treatment decreased oxidative neuronal stress by reducing MDA levels in the brains of Alzheimer rats. The same team also found that hydrogen-rich saline treatment decreased the levels of IL-1β, 8-OH-dG, JNK, and NF-kB in the amyloid-beta induced neural inflammation and oxidative rat model [45]. Lactulose, as a special class of carbohydrate, showed the protective effect on several animal models of injuries caused by oxidative stress through hydrogen gas production [16,17,18]. In this study, both lactulose and HRW treatments not only ameliorated the reduction of growth performance but also reduced the oxidative biomarkers levels in the serum and liver. Moreover, the activities of the CAT, total SOD, CuZn-SOD, and Mn-SOD levels were also improved in hepatic samples, demonstrating that the beneficial effects of lactulose and HRW on growth performance were partially mediated through their antioxidative property. The possible scientific explanations for the biological activities of lactulose in this study might be summarized as follows: (a) prebiotics compounds can decrease the bioaccessibility of mycotoxins, with a concentration-dependent behavior [46]; (b) the synthetic disaccharide lactulose acts as a prebiotic that enhanced growth performance of piglets [21]; and (c) dramatic endogenous biological molecule hydrogens can be produced by bacteria fermentation [47]. The similar protective effects between lactulose and HRW also may partially support our hypothesis that hydrogen gas was one factor contributing to the LAC’s biological activities in countering the mycotoxins side effects. This study provides data that might help to propose a novel explanation that endogenous hydrogen gas might contribute to the beneficial effect of prebiotics. Therefore, dietary supplementation of lactulose or drinking HRW might be used as a novel antioxidant against *Fusarium* mycotoxins-induced mycotoxicosis in swine production. However, it must be kept in mind that this study was performed in a controlled environment with a limited number of pigs. Further studies are warranted to analyze the therapeutic effects of HRW and lactulose for piglets fed with mycotoxins contaminated diets.

## 4. Conclusions

The present results indicated that consumption of *Fusarium* mycotoxin-contaminated diets led to growth depression, organ damage, and oxidative stress in female piglets. Hydrogen-rich water and lactulose protected against the *Fusarium* mycotoxins induced growth depression and oxidative stress. These findings partly support our original hypothesis that supplementation of hydrogen-producing prebiotic may improve the antioxidant capacity by increasing intestinal hydrogen production in female weaning piglets.

## 5. Materials and Methods

### 5.1. Preparation of Fusarium Contaminated Maize

*Fusarium graminearum* strain 2021 was kindly supplied by Prof. Ming-Guo Zhou, College of Plant Protection, Nanjing Agricultural University, Nanjing, China. The strain was cultured as described in a previous study [48]. Briefly, the strain was first cultivated on potato sucrose agar (PSA) at 25 °C for 7 days. The hyphae of fungi were obtained, inoculated in mung bean broth (MBB), and cultured with shaking (200 rpm/min at 25 °C) for 7 days for conidia production. Before inoculation, maize was soaked with water for 48 h and autoclaved. The conidia were inoculated to cool autoclaved maize at a concentration of 1 × 10^6^ conidia/kg. The *Fusarium* contaminated maize was incubated in plastic storage boxes for 30 days (temperature 15–25 °C and humidity 50–85%). The cool autoclaved maize not inoculated with conidia was used as control maize. Finally, control and maize with *Fusarium* mold were dried in an oven at 70 °C for 24 h, respectively.

### 5.2. Experimental Diets and Mycotoxins Analysis

*Fusarium* mycotoxins-contaminated maize and uncontaminated control maize were used at 50% at the expense of normal maize for the manufacturing of two experimental diets, respectively. The experimental diets were formulated according to the recommendations of the nutrient requirement of swine by National Research Council [49] and based on a previous study [41], with a minor modification to the vitamin and mineral premix. No antibiotic, hormone and preservatives were added to the diets. Table A1 shows the ingredients of the experimental diets used in this study.

*Fusarium* mycotoxins levels in the two experimental diets were analyzed as previously described [50]. Briefly, a 10 g diet sample was extracted with 25 mL of acetonitrile:water (80:20, *v/v*) at 180 rpm for 30 min. After centrifugation at 3000 rpm for 10 min, 0.5 mL supernatant was diluted with 1.5 mL of acetonitrile:water (80:20, *v/v*) and filtered through a nylon filter (13 mm in diameter and 0.22 μm pore size). Then, the filtered solution was analyzed by a high-pressure liquid chromatography/electrospray ionization–tandem mass spectrometry (LC–MS/MS) system consisting of an Agilent 1200 HPLC (Agilent Technology, Shanghai, China), an Agilent 6410B triple-quadrupole mass spectrometer (Agilent Technology, Shanghai, China), and an Agilent MassHunter Workstation running Qualitative Analysis version B.01.03 software (Agilent Technology, Shanghai, China, 2001) for data acquisition and analysis. No significant differences were found in nutrient composition between the NC and MC experimental diets (Table A1). Levels of the main *Fusarium* mycotoxins in 2 experimental diets are summarized in Table A2. The NC diet contained 221.10 μg/kg DON, 12.12 μg/kg 3-acetyl DON, 32.95 μg/kg 15-acetyl DON, and 266.26 μg/kg total DON, respectively. While the MC diet contained 825.46 μg/kg DON, 212.79 μg/kg 3-acetyl DON, 59.45 μg/kg 15-acetyl DON, 1097.99 μg/kg total DON, and 501.56 μg/kg ZEN, which were all significantly higher (*p* < 0.05) than in the NC diet (Table A2).

### 5.3. Animals

All experiments and protocols used in this study were approved by the Nanjing Agricultural University Institutional Animal Care and Use Committee (Certification No.: SYXK (Su) 2011-0036, 11 August 2015). A total of 24 clinically healthy female weaning piglets (Landrace × large × white) from 6 littles (4 pigs/little) were individually housed in pens (1.2 by 2.0 m) with 1 feeder and 1 nipple drinker. The piglets had ab libitum access to feed and water.

### 5.4. Experiment Design and Sample Collection

The piglets from the same little were randomly assigned to receive 1 of 4 treatments (total 6 pigs/treatment): uncontaminated basal diet (negative control (NC)), mycotoxin-contaminated (MC) diet, MC diet + lactulose (MC + LAC) and MC diet + hydrogen-rich water (MC + HRW). After a 6-day adaption period, each treatment fed their respective diet for 25 days. Piglets received two times/day (10:00 and 14:00, respectively) and 10 mL/kg BW/time for their respective treatments. Piglets in NC and MC groups were orally administrated with hydrogen-free water (HFW), MC + HRW group received hydrogen-rich water (HRW), and MC + LAC group received 500 mg/kg BW of LAC (dissolved in 10 mL of HFW). The hydrogen-rich water (Beijing Hydrovita Biotechnology Company, Beijing, China) was kept in 300 mL aluminum pouches and administered to piglets within 15 min after opened. The H_2_ concentration was 0.6 mM as measured by an H_2_ sensor (Unisense, Aarhus, Denmark). Each animal’s daily feed intake and weekly body weight were recorded. Amounts of HFW, LAC and HRW were dependent on the body weight and updated weekly.

On Day 21, plasma samples at fasting and two hours after different administrations were collected to detect the hydrogen levels. One piglet was removed from the MC, MC + LAC and MC + HRW groups fed with *Fusarium* mycotoxins contaminated maize due to the poor health condition. Therefore, five independent replicates from each group were used in this study. At the end of the experiment, 30 min after administration of different treatments, piglets were euthanized by an intramuscular injection of sodium pentobarbital (40 mg/kg BW). The serum and liver were sampled.

### 5.5. Serum Hormones and Antioxidant Assay

Serum hormone levels of ghrelin, PYY, and CKK were measured by commercial ELISA kit according to their instructions (Fangcheng Beijing Technology Co. Ltd., Beijing, China). Liver samples were homogenized as previously described [51]. Oxidant and antioxidant parameters in the serum and liver supernatants were analyzed using assay kit according to the manufacturer’s instructions (Nanjing Jiancheng, Nanjing, China).

### 5.6. Hydrogen Gas Measurement in Plasma and Liver Samples

Hydrogen levels in serum samples were analyzed using a hydrogen sensor (Unisense, Aarhus, Denmark). Samples of liver were prepared as previously described [52]. Briefly, piglets were euthanized with sodium pentobarbital and placed in supine position. An incision was made in the abdomen. Hydrogen microelectrode (diameter, 50 μm) was inserted into the liver at a depth of 500 μm.

### 5.7. Statistical Analysis

All statistical analyses were performed using the SPSS software (Version 18.0, SPSS Inc., Chicago, IL, USA, 2009). The differences among treatments were evaluated using one-way ANOVA followed by Tukey–Kramer test. Data were considered to be statistically significant if *p* < 0.05.

## Figures and Tables

**Table 1:** Effects of hydrogen-rich water and lactulose on growth performance in female piglets fed *Fusarium* mycotoxins contaminated diets ^1,2^.

Item	NC	MC	MC + LAC	MC + HRW	SEM	*p*-Value
BW, kg						
Day 0	7.66	7.88	7.76	8.00	0.20	0.947
Day 14	12.42	10.42	12.27	12.14	0.39	0.244
Day 25	18.78	14.44	17.70	17.48	0.61	0.056
ADG, kg/day						
Days 0 to 14	0.34 ^a^	0.18 ^b^	0.32 ^a^	0.29 ^a^	0.02	0.013
Days 14 to 25	0.58	0.36	0.49	0.48	0.03	0.055
Days 0 to 25	0.44 ^a^	0.26 ^b^	0.40 ^a^	0.38 ^a^	0.02	0.008
ADFI, g/day						
Days 0 to 14	0.58 ^a^	0.39 ^b^	0.53 ^a^	0.58 ^a^	0.03	0.010
Days 14 to 25	1.04 ^a^	0.66 ^b^	0.86 ^a,b^	0.87 ^a,b^	0.05	0.034
Days 0 to 25	0.79 ^a^	0.51 ^b^	0.67 ^a^	0.71 ^a^	0.03	0.017
G:F, g/g						
Days 0 to 14	0.59	0.46	0.60	0.51	0.02	0.060
Days 14 to 25	0.56	0.56	0.59	0.55	0.02	0.948
Days 0 to 25	0.57	0.53	0.60	0.53	0.02	0.358

^a,b^ Values with different letters within the same row are different (*p* < 0.05). ^1^ NC (negative control), basal diet; MC, *Fusarium* mycotoxins contaminated diets; MC + LAC, MC diet + lactulose treatment; and MC + HRW, MC diet + hydrogen-rich water treatment. ^2^ *n* = 5.

**Table 2:** Effects of hydrogen-rich water and lactulose on serum hormones levels in female piglets fed *Fusarium* mycotoxins contaminated diets ^1,2^.

Item	NC	MC	MC + LAC	MC + HRW	SEM	*p*-Value
Ghrelin (ng/L)	3743.92	3064.05	3533.69	3587.87	85.79	0.112
PYY (pg/mL)	740.09 ^b^	837.44 ^a^	729.94 ^b^	727.60 ^b^	13.77	0.003
CCK (ng/L)	178.29 ^b^	211.72 ^a^	176.26 ^b^	157.63 ^b^	7.65	0.007

^a,b^ Values with different letters within the same row are different (*p* < 0.05). ^1^ NC (negative control), basal diet; MC, *Fusarium* mycotoxins contaminated diets; MC + LAC, MC diet + lactulose treatment; and MC + HRW, MC diet + hydrogen-rich water treatment. ^2^ *n* = 5.

**Table 3:** Effects of hydrogen-rich water and lactulose on blood antioxidant capacity in female piglets fed *Fusarium* mycotoxins contaminated diets ^1,2^.

Items	NC	MC	MC + LAC	MC + HRW	SEM	*p*-Value
Total carbonyl (mg/mL)	0.78 ^b^	1.07 ^a^	0.84 ^b^	0.86 ^b^	0.04	0.047
8-OH-dG (ng/mL)	8.41 ^b^	11.97 ^a^	8.24 ^b^	8.14 ^b^	0.48	0.002
MDA (nmol/mL)	4.15 ^a,b^	4.79 ^a^	3.23 ^b^	3.66 ^a,b^	0.29	0.043
CAT (U/mL)	2.54	3.96	2.52	2.86	0.23	0.079
Total-SOD (U/mL)	76.06	77.43	77.02	73.69	1.11	0.669
CuZn-SOD (U/mL)	72.20	70.94	74.16	69.33	1.20	0.576
Mn-SOD (U/mL)	6.06	6.48	5.86	6.56	0.19	0.562

^a,b^ Values with different letters within the same row are different (*p* < 0.05). ^1^ NC (negative control), basal diet; MC, *Fusarium* mycotoxins contaminated diets; MC + LAC, MC diet + lactulose treatment; and MC + HRW, MC diet + hydrogen water treatment. ^2^ *n* = 5.

**Table 4:** Effects of hydrogen-rich water and lactulose on the liver antioxidant status in female piglets fed *Fusarium* mycotoxins contaminated diets ^1,2^.

Items	NC	MC	MC + LAC	MC + HRW	SEM	*p*-Value
Total carbonyl (mg/g wt liver)	1.64 ^a,b^	1.84 ^a^	1.58 ^b^	1.56 ^b^	0.04	0.028
Inhibiting hydroxyl radical (U/mg protein)	1979.50 ^b^	2444.81 ^a,b^	2777.68 ^b^	3140.79 ^a^	141.28	0.013
Protein carbonyl (nmol/mg protein)	5.63	5.26	5.91	6.52	0.25	0.369
Lipid peroxidation (μmol/g protein)	1.41 ^b^	2.09 ^a^	1.42 ^b^	1.41 ^b^	0.08	<0.001
MDA (nmol/mg protein)	1.55	1.28	1.16	1.24	0.05	0.061
CAT (U/mg protein)	24.60 ^a,b^	27.95 ^a^	21.21 ^b,c^	19.19 ^c^	0.93	<0.001
Total-SOD (U/mg protein)	206.61 ^a^	188.58 ^b^	212.62 ^a^	209.00 ^a^	3.20	0.023
CuZn-SOD (U/mg protein)	141.31 ^b,c^	137.12 ^c^	157.88 ^a^	153.58 ^a,b^	2.79	0.011
Mn-SOD (U/mg protein)	65.30 ^a^	49.45 ^b^	58.74 ^a,b^	57.42 ^a,b^	2.02	0.033
GSH-px (U/mg protein)	130.29	122.56	107.93	111.98	3.46	0.078
T-GSH (μmol/g wt liver)	1.84	1.80	1.80	2.09	0.11	0.801
GSSG (μmol/g wt liver)	0.59	0.51	0.66	0.57	0.03	0.199
Reduced GSH (μmol/g wt liver)	0.66	0.79	0.49	0.95	0.10	0.474

^a–c^ Values with different letters within the same row are different (*p* < 0.05). ^1^ NC (negative control), basal diet; MC, *Fusarium* mycotoxins contaminated diets; MC + LAC, MC diet + lactulose treatment; and MC + HRW, MC diet + hydrogen water treatment. ^2^ *n* = 5.

## Appendix A

**Table A1:** Ingredient composition and nutrient contents of control and experimental diets.

Item	NC ^1^ Diet	MC ^2^ Diet
Ingredients, %		
Normal corn	16.75	16.75
*Fusarium* toxins uncontaminated corn	44.50	-
*Fusarium* toxins contaminated corn	-	44.50
Soybean meal	15.79	15.79
Extruded soybean	10.0	10.0
Fish meal	5.0	5.0
Wheat bran	3.0	3.0
Soybean oil	1.74	1.74
Vitamin and mineral premix ^3^	1.0	1.0
Limestone powder	0.98	0.98
Calcium hydrogen phosphate	0.78	0.78
Salt	0.37	0.37
Lysine HCl (98%)	0.09	0.09
Total	100.00	100.00
Analyzed chemical composition ^4^		
DM, %	88.96	88.28
CP, %	20.11	20.4
Crude ash, %	4.70	4.89
Crude fiber, %	1.71	1.96
Ether extract, %	8.04	8.65
Calculated DE, ^5^ kcal/kg	3400.00	3400.00

^1^ NC, negative control (basal diet). ^2^ MC, mycotoxin-contaminated diet. ^3^ Provided, per kilogram of diet (as-fed basis) 55 mg Zn (ZnSO_4_), 30 mg Cu (CuSO_4_), 60 mg Mn (MnSO_4_), 120 mg Fe (FeSO_4_), 1 mg I (KI), 2 mg Co (CoSO_4_), 0.3 mg Se (Na_2_SeO_3_), 9000 IU vitamin A, 1800 IU vitamin D_3_, 40 IU vitamin E, 3 mg vitamin B_1_, 4.5 mg vitamin B_2_, 16 mg pantothenic acid, 10 mg vitamin B_6_, 0.08 mg vitamin B_12_, 28 mg niacin, 2 mg folic acid, 1.8 mg vitamin K_3_, 0.2 mg biotin, 800 mg choline chloride, and 100 mg vitamin C. The premix did not contain additional Cu, Zn, antibiotics, or probiotics. ^4^ Unless indicated otherwise. ^5^ Based on a DM content of 88%.

**Table A2:** Mycotoxins composition of basal and fusarium mycotoxins contaminated diet ^1,2^.

Mycotoxin (μg/kg)	NC	MC	SEM	*p*-Value
Deoxynivalenol (DON)	221.10	825.46 *	84.34	<0.01
3-acetyl DON	12.12	212.79 *	30.46	<0.01
15-acetyl DON	32.95	59.75 *	4.14	<0.01
Total DON	266.16	1097.99 *	116.30	<0.01
Zearalenone (ZEN)	9.61	501.56 *	69.82	<0.01
Nivalenol (NIV)	N.D	N.D	-	-
T-2	N.D	N.D	-	-

^1^ NC (negative control), basal diet; MC, mycotoxin-contaminated diet; Limit of detections: DON, 3-acrtyl and 15-acetyl DON are 20 ng/mL. ZEN, NIV and T-2 are 10 ng/mL. N.D, not detected; ^2^ *n* = 3. * Value between the NC and MC groups are different (*p* < 0.05).
